# Recent Advances in Deciphering Normal and Diseased Aortic Valve Biology Using Transcriptomic Technologies

**DOI:** 10.1111/jcmm.70835

**Published:** 2025-09-03

**Authors:** Monica Madalina Tucureanu, Ileana Manduteanu

**Affiliations:** ^1^ Institute of Cellular Biology and Pathology “Nicolae Simionescu” Bucharest Romania

**Keywords:** calcific aortic valve disease, multi‐omics, Phyisiology of aortic valve, therapeutic targets, transcriptomics

## Abstract

Calcific aortic valve disease (CAVD) is a growing global health burden, with no approved pharmacological treatments to date, indicating a substantial therapeutic gap and the need for deeper insight into its underlying mechanisms. Transcriptomic approaches, particularly RNA sequencing (RNAseq) and single‐cell sequencing (scRNAseq), are emerging as powerful tools for unravelling the complex biology of the aortic valve (AV) in both normal and diseased states. This review summarises recent advances in our understanding of AV structure and function, with emphasis on valvular cell plasticity, heterogeneity and intercellular interactions—especially between valvular endothelial cells (VECs) and monocytes under physiological and pathological conditions. We further underscore the importance of characterising baseline molecular and cellular processes in the healthy AV to properly interpret disease‐associated alterations. By integrating recent transcriptomic findings, this review identifies key molecular targets and prioritises them for validation through wet‐lab experiments, with the ultimate goal of informing the development of effective therapies for CAVD.

**Abbreviations**
AEBP1: AE Binding Protein 1
AFAP1: Actin Filament Associated Protein 1
ALDH1A2: Aldehyde Dehydrogenase 1 Family Member A2
ALP: Alkaline phosphatase
ANGPTL: Angiopoietin‐like
ANXA1: Annexin A1
AV: Aortic valve
AVD: Aortic valve disease
BCL10: B‐cell lymphoma/leukaemia 10
BMP: Bone morphogenetic protein
CAVD: Calcific aortic valve disease
CCL20: C‐C Motif Chemokine Ligand 20
CD28: Cluster of differentiation 28
CD29: Cluster of differentiation 29
CD36: Cluster of differentiation 36
CD3D: Cluster of differentiation 3D
CD40: Cluster of differentiation 40
CD44: Cluster of differentiation 44
CD45: Cluster of differentiation 45
CD59: Cluster of differentiation 59
CD73: Cluster of differentiation 73
CD8: Cluster of differentiation 8
CDAN1: Codanin 1
CELSR2: Cadherin EGF LAG Seven‐Pass G‐Type Receptor 2
CEP85L: Centrosomal Protein 85 Like
CircHIPK3: Circular RNA Homeodomain Interacting Protein Kinase 3
CMSS1: Cms1 Ribosomal Small Subunit Homologue
CNP: 2′,3′‐Cyclic Nucleotide 3′ Phosphodiesterase
CTHRC1: Collagen Triple Helix Repeat Containing 1
DEG: Differentially expressed genes
DKK2: Dickkopf WNT Signalling Pathway Inhibitor 2
EC: Endothelial cell
ECM: Extracellular matrix
EGFR: Epidermal growth factor receptor
EMT: Epithelial–mesenchymal transition
EndMT: Endothelial‐to‐mesenchymal transition
FABP4: Fatty acid binding protein 4
FABP5: Fatty acid binding protein 5
FADS: Fatty acid desaturase
FAK: Focal adhesion kinase
FES: FES proto‐oncogene
FGF‐3: Fibroblast Growth Factor 3
FGF‐3R: Fibroblast Growth Factor Receptor 3
FMT: fibroblast‐to‐myofibroblast transdifferentiation
FNDC1: Fibronectin Type III Domain Containing 1
FOS: FOS proto‐oncogene
FTO: Fat mass and obesity associated protein
GWAS: Genome‐Wide Association Study
H19: H19 Imprinted Maternally Expressed Transcript
HMOX1: Heme oxygenase 1
HSPA6: Heat Shock Protein Family A Member 6
IL1F9: Interleukin 1 Family Member 9
IL1RL1: Interleukin 1 Receptor‐Like 1
IL6: Interleukin 6
KEGG: Kyoto Encyclopedia of Genes and Genomes
LDL: Low‐density lipoprotein
LDLR: Low‐density lipoprotein receptor
lncRNA: Long non‐coding RNA
LPA: Lipoprotein(a)
LPL: Lipoprotein lipase
LUM: Lumican
MAOA: Monoamine oxidase A
MDK: Midkine
MECOM: MDS1 and EVI1 complex locus
MHC: Major histocompatibility complex
MT1A: Metallothionein 1A
MUC4: Mucin 4
MXRA5: Matrix Remodelling Associated 5
NAV1: Neuron Navigator 1
NMR: Nuclear magnetic resonance
NO: Nitric oxide
NOTCH: Notch homologue
NRBP1: Nuclear Receptor Binding Protein 1
OPG: Osteoprotegerin
PALMD: Palmdelphin
PCSK9: Proprotein Convertase Subtilisin/Kexin Type 9
PI3: Peptidase Inhibitor 3
PI3K: Phosphoinositide 3‐kinase
piRNA: Piwi‐interacting RNA
PKC: Protein kinase C
PLPP3: Phospholipid phosphatase 3
PPARγ: Peroxisome proliferator‐activated receptor gamma
PTH: Parathyroid hormone
RAD9A: RAD9 checkpoint clamp component A
RNAseq: RNA sequencing
ROCK1: Rho‐Associated Coiled‐Coil Containing Protein Kinase 1
RPL17: Ribosomal protein L17
RUNX2: Runt Related Transcription Factor 2
scRNAseq: Single‐cell RNA sequencing
SELE: Selectin E
SLMAP: Sarcolemma associated protein
SMAD9: SMAD Family Member 9
SOX4: SRY‐Box Transcription Factor 4
SPARC: Secreted protein, acidic, rich in cysteine
SPP1: Secreted Phosphoprotein 1 (Osteopontin)
TEX41: Testis Expressed 41
TGFB: Transforming growth factor beta
THBS1: Thrombospondin 1
THBS2: Thrombospondin 2
TWAS: Transcriptome‐Wide Association Study
TWEAK: TNF‐Related Weak Inducer of Apoptosis
TWIST1: Twist‐related protein 1
VASP: Vasodilator‐stimulated phosphoprotein
VDSC: Ventricular derived stem cells
VEC: Valvular endothelial cell
VIC: Valvular interstitial cells
VTN: Vitronectin
Wnt: Wnt family member
XCR1: X‐C Motif Chemokine Receptor 1

## 1 Introduction

The aortic valve (AV) ensures unidirectional blood flow from the left ventricle into the aorta. Each AV leaflet comprises three layers—fibrosa, spongiosa and ventricularis [1]—and communicates with surrounding tissues through humoral and mechanical stimuli [2]. The AV surfaces are lined by valvular endothelial cells (VECs), which regulate vascular tone, inflammation, thrombosis and remodelling [3]. These cells are contiguous with the myocardial and aortic endothelium and originate from embryonic cardiac cushion endothelial cell populations. Valvular interstitial cells (VICs) are the predominant cell type, maintaining tissue homeostasis and structural integrity [4]. Found across all 3 layers, VICs are fibroblast‐like cells and arise from various embryonic sources, including the endocardial cushions, neural crest and epicardium [5]. The valvular stroma also contains a small population of resident macrophages, with occasional smooth muscle cells and immune cells.

Aortic valve diseases (AVD)—especially calcific aortic valve disease (CAVD)—are progressive and age‐associated, posing major health challenges worldwide [6]. Currently, no pharmacological interventions exist to prevent or reverse the progression of CAVD, indicating the need for deeper mechanistic insights. A clearer understanding of the molecular and cellular pathways driving AVD could lead to new therapeutic strategies, including those based on tissue engineering and regenerative medicine. Elucidating these mechanisms is also vital for early diagnosis and improving patient outcomes and quality of life.

Transcriptomic approaches, particularly bulk RNA sequencing (RNAseq) and single‐cell sequencing (scRNAseq), have emerged as indispensable tools in AV research. These techniques offer unprecedented insights into gene expression dynamics, cellular heterogeneity, intercellular interactions and molecular targets. RNAseq provides a comprehensive view of gene expression, splicing variants and mutations across different conditions, offering a detailed genetic map of AV biology [7]. Bulk RNAseq captures differential gene expression patterns at the tissue or cell population level [8], while scRNAseq identifies discrete cellular subpopulations across normal and disease states, thereby enhancing a comprehensive understanding of the cellular characteristics and mechanisms underlying the disease [9].

**FIGURE 1:**
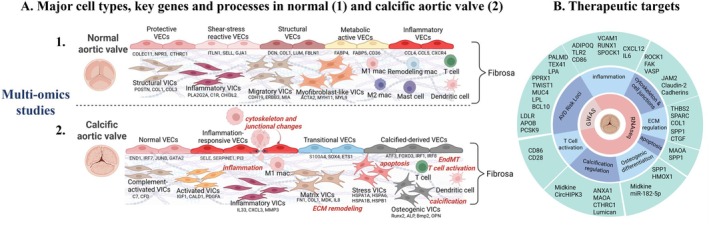
Schematic representation of the molecular and cellular landscape of normal and calcific aortic valve (A), and potential therapeutic targets in CAVD (B) as explored by omics approaches. A1. Cellular heterogeneity and specific biomarkers in normal aortic valve. A2. Cell types, molecular signatures and key driver processes in CAVD. (B) Putative therapeutic targets and associated key processes in CAVD. (Created with BioRender).

This review synthesises recent advances in AV biology and CAVD from omics and multi‐omics studies. By highlighting the molecular and cellular heterogeneity of the AV and the key regulatory pathways involved, we aim to identify promising therapeutic targets and propose future directions for translational research and therapy development. A graphical summary of this review is shown in Figure 1 (created with Biorender). The cell types, key genes and processes in the normal aortic valve (panel A1) and in the calcific aortic valves (panel A2) as well as possible molecular therapeutic targets (panel B) revealed by multi‐omics are illustrated.

## 2 The Landscape of Normal Aortic Valve Structure and Function

The AV is far more than a passive structure—it is a complex and dynamic system that operates through active communication between its cellular and molecular components. This idea of the intricate nature of AV function was first emphasised in 1999 by Yacoub et al. [10] and this concept shifted our understanding of AV physiology from a simplistic model to one that recognises its complexity and adaptability. scRNA studies on normal AV specimens revealed the transcriptomic landscape of a healthy AV [11, 12] and have shaped the current understanding of the AV's proper functioning, which is governed by a dynamic interplay of molecular mechanisms that involve a variety of cellular processes, signalling pathways and ECM components that work together to maintain the structural and functional integrity of the AV. These new data will play a crucial role in shaping and guiding the development of future therapies for aortic valve conditions.

### 2.1 Cellular Heterogeneity of the Normal Aortic Valve

#### 2.1.1 Valvular Endothelial Cells (VEC)

Endothelial cells lining the aortic valve (VECs) are critical for maintaining valvular homeostasis. They regulate vascular tone, inflammation, thrombosis and remodelling and respond to shear‐stress by translating mechanical stimuli into biological responses [13]. VECs secrete extracellular matrix (ECM) components and signalling molecules that support the structural integrity and proper mechanical function of the valve. Transcriptomic studies have identified functionally distinct VECs across species (human and mouse) (Table 1), as well as unique transcriptional profiles in swine VECs compared to aortic endothelial cells [14]. These include differences in growth regulation, transcriptional activity, gene expression, growth factor response and immunogenic potential. However, the specific functions of these cells require validation by functional assays, as transcriptomic data alone provide inferred roles. In VECs, increased activity of transcription factors associated with proliferation such as JunD and PKC, and elevated expression of mitogenic factors FGF‐3 and FGF‐3R, suggest a potential autocrine loop that promotes their proliferation [14].

**TABLE 1:** Cellular heterogeneity in normal AV.

Species	Cell type	Defined clusters	Specific markers	Samples	Accession ID	Method	References
Mouse (C57/Bl6)	VEC	Traditional VEC	SCX, EMCN, NRP1, CPE, BGN, EDN1	Healthy AV from postnatal day 1	GSE269604	scRNAseq (10× Genomics)	[11]
		Coapt VEC	CRIP1, PRNP, HAPLN1, FXYD5, WNT9B, MTS, PTHLH, PODXL, PI16				
		Lymph VEC	LSAMP, ENPP2, FBN2, PROX1, EBF1, PDGFRA, POSTN, RDX, TSHZ2				
		PND1 VEC	VEGFC, ARL15, JAG1, GJA5, COL8A1, MGP, MECOM				
	VIC	Primitive VIC	SCX, MSX1, SOX9, TBX20, ABI3BP, DKK3				
		Remodelling VIC	COL4A1, PTN, COL14A1, ABCA8A, MATN2, MFAP5, GSN, FBN1, COL24A1				
		Bioactive VIC	COL1A1, COL1A2, SPARC, FMOD, FBLN2, CFH, GAS6, IGFBP3, SERPING1, BICC1, ANGPTL7, MGP				
Human	VEC	Protective VEC	COLEC11, NPR3, HLA‐DPA1, HLA‐DRA, PLA2G2A, CTHRC1, HLA‐DRB1	Adult cardiac valves with end‐stage heart failure	GSE213128	scRNAseq (10× Genomics)	[12]
		Shear‐stress reactive VEC	ITLN1, CYP1B1, SELL, PLAT, LIFR, CXCL2, GJA1				
		Structural VEC	DCN, COL1A2, LUM, FBLN1, COL1A1				
		Metabolically active VEC	FABP4, FABP5, CD36, H19, CLDN5				
		Inflammatory VEC	CCL4, CCL5, CXCR4				
	VIC	No‐associated VIC	HES4, CCL2, CXCL12, CA3, RARRES2, GUCY1A1				
		Silent VIC	ANGPTL7, CCN5, SFRP1, CD34				
		Structural VIC	POSTN, COL1A1, PTN, COL1A2, IGF1, ASPN, COL3A1, SPARC, IGF1				
		Pro‐inflammatory VIC	PLA2G2A, C1R, CRLF1, CHI3L2				
		Migratory VIC	SERPINE2, CDH19, ERBB3, MIA, S100B, S100A1				
	
	Myofibroblast‐like VIC	CCL21, FABP4, DES, MYH11, TFF3, MYL9, ACTA2				
	Myeloid cells	M1‐like macrophage					CCL3L1, IL1B, CXCL8, TNF, NLRP3, HLA‐DPA1, CCL4L2, RGS1, HLA‐DQA1, CCL3, HLA‐DQA2, HLA‐DPB1
		M2‐like macrophage					CCL18, MARCO, GPNMB, CTSB, ACP5, PLTP, LGMN, FTL, CTSD
		Remodelling macrophage					MGP, CLU, COL1A1, DCN, COL1A2, COL3A1
		Monocyte‐derived FCN1+ macrophage					S100A8, EREG, THBS1, LYZ, VCAN, S100A9, FCN1, S100A12, CD52
Dividing macrophage		TOP2A, TUBB, MKI67, HMGN2, CKS1B, HIST1H4C, STMN1, HMGB2, TUBA1B, HMGB1				

*Note:* The diversity of cell types present in the normal AV and their defined clusters across various species. For each species (Mouse and Human), specific cell types are listed along with their respective defined clusters and specific markers that characterise these clusters. Samples are described based on their origin. Accession IDs link to genomic datasets for further exploration of the underlying genomic information. References are provided for data source.

ScRNAseq in mice across embryonic, postnatal and adult stages has revealed that while VECs maintain stable molecular profiles, gene expression changes significantly between embryonic and ageing stages [15]. With age, nitric oxide production, cell repair and proliferation decline: VEC density also decreases, leading to greater permeability and reduced intercellular interactions. A unique subpopulation defined as ‘postnatal day1‐VEC’, expressing genes involved in migration and proliferation, appears critical for early postnatal maturation [11]. These transcriptomic studies might help to understand the precise regulation of cellular processes, beginning in embryonic stages and continuing postnatally as the valve matures.

In healthy human valves, five VEC populations have been identified: Protective, shear‐stress‐responsive (also expressing endothelial‐to‐mesenchymal transition genes), structural, metabolically active and inflammatory VECs [12]. These molecular signatures could help explain valve susceptibility to disease and aid in the development of therapeutic strategies. Interestingly, a sub‐population of aortic ECs shares gene expression with metabolically active VECs (characterised by FABP4, FABP5 and CD36) indicating a role in lipoprotein metabolism [16]. VECs are thus key players in regulating lipid metabolism under normal conditions, as evidenced by the enrichment of CD36+ VECs and a high PPARγ expression, promoting LDL uptake and modulating inflammation [17].

#### 2.1.2 Valvular Interstitial Cells (VICs)

VICs are the predominant cell type in the valvular tissue and are essential for maintaining ECM homeostasis and structural integrity. In adults, quiescent VICs help prevent excessive remodelling. Distinct VIC phenotypes—including activated, progenitor and osteoblastic—support tissue repair and prevent pathological changes like fibrosis and calcification, highlighting their central role in maintaining the healthy function of the aortic valve [18, 19].

VICs interact dynamically with VECs and immune cells to maintain valve structure and function. Postnatal VICs show leaflet‐specific expression of complement components, ECM proteins and osteogenic genes, with distinct subpopulations involved in matrix remodelling and morphogenesis, as shown in C57Bl6J mouse models [11, 20]. Combining scRNAseq with lineage tracing and knockout approaches, Adamts19 was identified as a marker of VIC maturation in mice [21], and Pitx2 as a regulator of endocardial cell differentiation [22].

VICs exhibit remarkable plasticity, transitioning between quiescent, activated, and myofibroblastic states in response to various stimuli [23]. Quiescent VICs maintain ECM stability through non‐contractile activity and focal adhesions [18]. scRNAseq of healthy human cardiac valves identified new VIC populations beyond the traditional quiescent/activated classification: three quiescent (NO‐associated, silent and structural) and three activated VIC phenotypes (pro‐inflammatory, migratory and myofibroblast‐like), each with specific gene markers. The most abundant quiescent VICs participate in NO signalling, offering protection against fibrosis and calcification, while migratory VICs were the most prevalent among activated cells. Myofibroblast‐like VICs were more abundant in the human aortic valve compared to mitral, pulmonary or tricuspid valves, possibly explaining its higher susceptibility to calcification and ECM remodelling. VICs also interact with VECs and myofibroblasts via pathways such as CD40, BMP, and TWEAK, promoting migration, inflammation and calcification [12].

#### 2.1.3 Resident Immune Cells

Immune cells constitute approximately 8% of total valve cells during development, with macrophages representing over 75%, alongside dendritic and T cells [20]. Tissue‐resident macrophages, which arise during embryogenesis and persist into adulthood, are the most abundant macrophage population and play key roles in immune surveillance and tissue homeostasis [24, 25]. Postnatally, they are recruited from the closing ductus arteriosus, integrate into the tunica intima, and proliferate in situ [26]. These macrophages help clear apoptotic cells during mid‐late gestation [27] and their depletion leads to increased apoptotic bodies and valve thickening, highlighting their importance in clearance and remodelling prevention [28].

Myeloid cells, the second largest immune population in the AV, include M1‐/M2‐like, remodelling, monocyte‐derived, and dividing macrophages in non‐diseased human AV [12].

Murine valve development studies have also identified T cells, mast cells and dendritic cells. T cells expressing CD24a, TRBC1, CD3d and CD8a help suppress excessive inflammation and promote immunologic tolerance [20, 29]. Dendritic cells, expressing KLRB1 and XCR1, may provide antigen surveillance [20]. They act as antigen‐presenting cells and are capable of cross‐presenting antigens to MHC class I to CD8+ T cells, suggesting their involvement in immune response regulation [30, 31]. Mast cells, expressing KIT, CPA3 and TPSAB1, likely play important but not fully characterised roles in valve maturation [20].

### 2.2 Cellular Interactions in Normal Aortic Valve Function

Once considered passive linings, VECs are now recognised as active regulators protecting against metabolic, mechanical and inflammatory stressors. Their homeostasis function depends on communication with other VECs, resident cells, blood components, and the ECM to maintain vascular integrity and function. Disruptions of these interactions compromise barrier integrity, increase permeability and heighten inflammation risk [32]. Endothelial barrier function relies on intercellular junctions, cytoskeletal organisation and focal adhesions anchoring cells to the ECM. Together, these components regulate tissue permeability.

VEC‐VIC interactions are critical for ECM composition, structural integrity and adaptation to hemodynamic forces [33]. VECs in adult heart valves can replenish VICs via endothelial‐to‐mesenchymal transition (EndMT) and reshape leaflets [34]. Transcriptomic studies suggest VEC‐VIC communication occurs through the endothelin signalling pathway and, in transformed VECs, via the angiopoietin‐like (ANGPTL) signalling pathway [35]. However, further studies are needed to fully elucidate these complex interactions.

Our RNAseq data show that monocyte interaction with VEC under normal conditions triggers transcriptomic changes associated with cytoskeleton disorganisation, altered junctional complexes, and modified ECM adhesion, ultimately increasing endothelial permeability [3]. Differentially expressed genes (DEGs) were enriched in KEGG pathways including actin cytoskeleton regulation, PI3K‐Akt signalling, focal adhesion, tight and adherens junctions and leukocyte transendothelial migration. Validation studies confirmed the upregulation of VASP and ROCK1, activation of PI3K, and downregulation of FAK, paxillin, Claudin‐5 and VE‐cadherin, proving novel evidence that endothelial barrier regulation is closely tied to VEC‐monocyte interactions.

Overall, transcriptomic technologies have revealed unprecedented details of aortic valve cellular heterogeneity, identifying functionally distinct cell types with unique molecular signatures. These findings provide critical insights into cellular interactions, mechanisms of homeostasis and potential vulnerabilities to disease. Understanding this complexity will guide future strategies to restore valve integrity and prevent pathological remodelling. A summary of healthy AV cellular heterogeneity is presented in Table 1.

## 3 Molecular Insights From Omics Technologies in Calcific Aortic Valve Disease

### 3.1 Disease Overview and Omics Contributions

CAVD is the most common form of aortic valve disease and progresses through distinct pathological stages [5, 36]. Early‐stage disease comprises endothelial dysfunction involving key processes like inflammation and cytoskeleton and junctional changes, and manifests as aortic sclerosis, characterised by leaflet thickening due to focal lipid infiltration and progresses to ECM remodelling, endothelial‐to‐mesenchymal transition, apoptosis of valvular cells, T cell activation and calcification (Figure 1, panel A2). Clinical risk factors include dyslipidemia, the metabolic syndrome, hypertension, smoking and elevated BMI [37, 38]. However, once aortic sclerosis is present, the risk factors driving progression to AV stenosis are unclear, and individual risk factors are not consistently associated with calcification progression [39]. The key pathological processes in CAVD and the underlying pathology have been reviewed elsewhere [5, 40].

Transcriptomic technologies, particularly RNAseq, allow simultaneous assessment of thousands of genes, enabling the identification of DEGs and dysregulated pathways involved in CAVD progression. Coupling transcriptomics with machine learning further improves our understanding of valvular pathophysiology and enables predictive modelling of calcification risk [41].

These approaches help identify key genes, biological pathways and potential biomarkers that play a role in how the disease develops, is diagnosed, and might be treated in the future.

### 3.2 Cellular Heterogeneity in CAVD

Valve cells exhibit regional and functional heterogeneity that influences disease susceptibility [42]. VECs display distinct gene expression profiles on the fibrosa and ventricularis sides of the valve, particularly affecting calcification inhibitors such as osteoprotegerin (OPG), C‐type natriuretic peptide (CNP) and parathyroid hormone (PTH) [43]. VICs also show regional variability, with fibrosa‐derived VICs being more calcification‐prone [42].

Recent scRNA studies have generated detailed cellular and molecular atlases of the human AV in CAVD [44]. Fourteen distinct cell types were identified, including three heterogeneous VIC subsets marked by FOS, HSPA6 and SPARC. Six novel valve‐derived stromal cell (VDSC) populations were found—enriched in CAVD tissue—and characterised by expression of LUM, SOX4, CCL20, MT1A, RPL17, CMSS1 and PI3 [44]. Pseudotime trajectory analysis revealed VEC‐to‐mesenchymal differentiation trajectories, implicating endo‐MT in lesion progression and identifying SELE, IL1RL1 and SPARC as key transitional markers.

Another scRNA study identified diverse functional valvular cell types and mechanisms of cell–cell communication within the human AV microenvironment in CAVD [35]. VICs were categorised into subtypes: activated (myofibroblast‐like), complement‐activated, inflammation‐associated, matrix‐producing, lipid‐associated and stress‐responsive VICs. VECs were also stratified into normal, calcified and transitional (mesenchymal‐like) VECs. Immune cells—macrophages, T‐cells and dendritic cells—were present and secreted cytokines that activated VICs. Notably, matrix VICs secreted midkine (MDK), which suppressed VIC calcification. In vitro, MDK inhibited osteogenic differentiation and reduced expression of osteogenic markers RUNX2 and ALP [35].

A novel population of disease‐driver VICs (DDP‐VICs) was identified by combined scRNAseq and proteomics in human calcified valves. These cells, marked by CD44^++^CD29^+^CD59^+^CD73^+^CD45^−^ were enriched near calcification sites and demonstrated multi‐lineage differentiation potential [45]. MAOA and CTHRC1 were identified as potential regulators of CAVD, with in vitro inhibition attenuating VIC calcification. Another study identified a unique lumican‐positive VIC population in CAVD patients [43]. The pro‐calcification role of LUM was confirmed on the in vitro, ex vivo, in vivo level and ApoE^−/−^/LUM^−/−^ double knockout mice. Lumican (LUM), a ubiquitous ECM proteoglycan, enhanced cellular glycolysis, leading to lactate accumulation and subsequent H3 histone lactylation, increasing RUNX2 and BMP2 gene expression and accelerating calcification. Knockout of LUM in ApoE^−/−^ mice reduced AV calcification [46].

Table 2 and Figure 1 panel A2 summarises the cellular heterogeneity observed in human and mouse AV tissues in CAVD. These data offer a much‐needed database of valvular cell transcriptomes, supporting ongoing efforts to identify valvular cell–mediated mechanisms underlying valve pathology.

**TABLE 2:** Cellular heterogeneity in AV disease.

Species	Cell type	Defined clusters	Specific markers	Samples	Accession ID	Method	References
Human	VEC	2 clusters	SELE, IL1RL1, SERPINE1, SLC7A2, PI3	Adult AVs from two healthy controls and four CAVD patients	PRJNA562645	scRNAseq (10× Genomics)	[44]
			SELE, SERPINE1, PI3, CFB, CTSB				
	VIC	3 clusters	FOS, PDK4, PLIN2, CRYAB, TNFAIP6				
			HSPA6, HSPA1A, HSPA1B, GADD45B, DNAJB1				
			COL1A1, COL3A1, SPARC, COL1A2, FN1				
	Leukocyte	Monocytes	CXCL13, PTX3, CXCL2, CCL2, CXCL1				
		Macrophages	CCL3L1, CXCL5, HLA‐DRA, CCL4, CCL3				
		Lymphocytes	IGJ, CD7, TMSB4X, CYBA, HIST1H4C				
	Stromal cells	6 clusters	LUM, IGFBP6, ADIRF, HLA‐B, BGN				
			SOX4, STC1, HNRNPH1, INHBA, ITGB1				
			CCL20, CXCL1, SEC61G, G0S2, IL8				
			MT1A, SRM, THAP2, ID4, G0S2				
			RPL17, IL33, METAP2, EIF3J, PTGDS				
			CMSS1, WBP5, ATP1B1, INHBA, SOX4				
Human	VECs	Normal‐derived VECs	END1, IRF7, JUNB, GAT/A2, ELF2, PGR, RBPJ, HDAC2	Adult AVs from two healthy controls and four CAVD patients	PRJNA562645	scRNAseq (10× Genomics)	[35]
		Calcified‐derived VECs	IRF1, CEBPD, NFκB1, FOSL1, IRF8, ATF3, FOXO3, ATF4, LRRFIP1, CDH2				
		Transitional VECs	MMP1, FN1, S100A4, KLF3, JUND, SOX4, ETS1				
	VICs	Activated VIC	IGF1, CALD1, PDGFA, IGFBP4				
		Complement‐activated VIC	C7, CFD				
		Inflammation‐associated VIC	IL33, CXCL3, MMP3				
		Matrix VIC	FN1, COL1A1, COL1A2, MDK, IL8				
		Lipid‐associated VIC	GPX3, APOE, FABP5, FRZB				
	
		Stress VIC	HSPA1A, HSPA6, HSPA1B, HSPB1				
	Unknown cluster	REL, THBS1, FBLN5					
	Immune cells	Macrophages	IL1B, CCL3, MMP9				
		T cells	CD3D, CD7, IFITM1				
		Calcific‐derived T cells	INHBA, IL11, IL6, TNFRSF11B				
Dendritic cells		CCR7, HLA‐DBP1, IDO1					
Human	VIC	Disease‐driver VICs	CD44, CD29, CD59, CD73, CD45 low	Isolated VICs from CAVD patients	GSE194180	scRNAseq (Illumina NextSeq)	[45]
Mouse	VEC	5 clusters: CD36+, FGFR3+, PROX1+, PDPN+, EDN1+	PECAM1, TEK, EMCN, CDH5	AVs from adult ApoE^−/−^ and Ldlr^−/−^ mice	GSE180278, GSE205587, GSE206927	scRNAseq (Illumina HiSeq2500)	[17]
	VIC	4 clusters: SPP1+, Meox+, ID4+, IRF7+	VIM, COL1A1, COL1A2, DCN				
	Leucocytes	Macrophages	ADGRE1, CSF1R, ITGAM, FCGR1, LYZ2				
		CDK1+ Macrophages	CDK1, CCNA2, MKI67				
		Dendritic cells	ZBTB46, ITGAX, FLT3, CD209A, XCR1				
		T cells	CD3D, CD3E, CD28, CD3G, CD2				
		B cells	CD79A, CD79b, MS4A1				

*Note:* The diverse cell populations identified in AV disease across human and mouse. For each species, specific cell types are categorised into defined clusters. Each cluster is associated with specific markers used for cellular identification. The sources of samples are indicated, highlighting those collected from pathological conditions. Accession IDs to genomic datasets for further investigation are documented, along with references for sourcing original data.

### 3.3 Transcriptomic Insights for Therapeutic Targets Identification

Multi‐omics approaches—integrating genomics, transcriptomics, proteomics, metabolomics and epigenomics—have expanded our understanding of CAVD mechanisms [47, 48]. These technologies enable the identification of biomarkers and therapeutic targets, facilitate early diagnosis and improve prognostic accuracy.

A core *circRNA/lncRNA‐miRNA‐mRNA network* has been identified in CAVD, highlighting the interaction of hsa‐circ‐0073813/hsa‐circ‐0027587, hsa‐miR‐525‐5p, and hub genes SPP1, HMOX1 and CD28—all upregulated in CAVD [49]. Immune profiling revealed increased M0 macrophages and memory B cells and decreased M2 macrophages and naive B cells, correlating with the upregulation of these genes. SPP1 is crucial in cell proliferation, apoptosis and migration [50, 51]; HMOX1 promotes osteogenic differentiation [52]; and CD68, a T‐cell costimulatory molecule, contributes to inflammation [53, 54].

CircHIPK3, downregulated in CAVD, was shown to suppress osteogenic markers (ALP, BMP‐2, RUNX2 and osteocalcin), calcium deposition and ALP activity. Its overexpression in mice reduced AV calcification and valve stenosis, leaflet thickening, calcium deposition and BMP‐2 expression [55]. In contrast, miR‐182‐5p was upregulated and suppressed Dkk2 expression, activating Wnt/β‐catenin signalling.

Other regulatory axes identified include LINC00702‐miR‐181b‐5p‐SPP1, linked to immune cell infiltration differences between calcified and non‐calcified valves [56].

*PiRNAs (piwi‐interacting RNAs), particularly has‐piR‐25624 (ACVAPIR)*, contribute to the pathogenesis of CAVD [57]. piRNAs are the largest class of ncRNAs, playing a crucial role in epigenetic regulation and transposon silencing in eukaryotic organisms [58]. AVCAPIR inhibits FTO (fat mass and obesity‐associated protein), blocking its N‐methyladenosine demethylase activity and enhancing CD36 mRNA stability and thus its binding partner PCSK9, a pro‐calcific gene, accelerating CAVD progression [57].

*Genome‐Wide Association Studies (GWAS)* have identified CAVD risk loci including: LPA, IL1F9 [59], PALMD, TEX41 [60, 61], IL6 and ALPL [62, 63], but functional validation of these loci is still ongoing.

23 genome‐wide significant lead variants in the GWAS, representing 17 unique genomic regions were identified in a multi‐ancestry GWAS and gene association study [64]. Of the 17 unique regions, 14 were significant in replication, representing 11 unique genomic regions. Five replicated genomic regions were previously known as AVD risk loci: PALMD, TEX41, IL6, LPA, FADS [59, 60, 61, 62, 65]. Six replicated genomic regions were novel: CEP85L, FTO, SLMAP, CELSR2, MECOM, CDAN1.

Combining GWAS with gene expression from 233 human aortic valve tissues, PALMD (palmdelphin) was identified as a new susceptibility gene for calcific AVS [61].

*Transcriptome‐Wide Association Studies (TWAS)* identified genes that were strongly associated with aortic stenosis [66]. The following genetic loci were identified: PALMD and NAV1, both previously associated with CAVD; PPRX1 and TWIST1, transcription factors involved in FMT and EMT, respectively [67, 68]; MUC4, involved in cardiac valve development [69]; ATP13A3 and ALDH1A2 enzymes; SMAD9, involved in TGFβ signalling [70]; LPL, LDLR, regulators of blood lipids [71]; and novel loci BCL10, RADA9, NRBP1, FES and AFAP1 that require further investigation in CAVD. The study revealed a strong genetic correlation between CAVD and several cardiovascular traits, including circulating lipids, blood pressure, diabetes and other cardiovascular diseases [66].

Although previous studies have reported the identification of PALMD via GWAS, its connection with transcriptomic approaches provides more compelling evidence for prioritising PALMD as a candidate gene and a potential driver in the development of CAVD.

*Epigenetic changes*, such as DNA methylation and histone modifications, also contribute to CAVD. Examples include methylation‐dependent regulation of 5‐lipoxygenase [72]. Similarly, hypermethylation of EGFR was linked with CAVD development in mice [73]. Hypomethylation of the H19 promoter region correlates with enhanced osteogenesis and NOTCH1 inhibition, contributing to CAVD pathology [74]. Notch1 promoter methylation promotes osteogenic differentiation of VICs [75]. The hypomethylation of the PLPP3 enhancer impacts lysophosphatidic acid metabolism [76].

*Mass spectrometry‐based proteomics* has revealed layer‐specific and stage‐specific protein changes in calcified valves. ANXA1 was found in extracellular vesicles and contributes to calcification [77, 78]. The identification of ApoC‐III as a promoter of calcification, through mitochondrial dysfunction, emerged from targeted proteomics studies combined with RNAseq [79]. Nuclear magnetic resonance (NMR) and mass spectrometry of plasma and urine samples from CAVD patients identified changes in lipid and amino acid metabolism [80, 81].

The investigation of proteomic architecture of valvular ECM identified AEBP1, FNDC1, MXRA5, THBS1, THBS2, VTN in calcified valves and AEBP1, FNDC1, SPP1 in fibrotic valves. Network analysis revealed strong connections between metabolic markers and ECM proteins. Two ECM proteins were proposed as potential biomarkers for aortic stenosis: FNDC1, potentially playing a role similar to fibronectin in osteoblast activity, and MXRA5, known to have anti‐fibrotic and anti‐inflammatory properties [82].

*Gene editing*, such as CRISPR‐Cas9, offers new avenues for modelling CAVD and potentially correcting pathogenic variants. In studies involving induced pluripotent stem cells (iPSCs), CRISPR was used to introduce specific genetic variants and assess their impact on cardiomyocyte function [83].

Table 3 and Figure 1 panel B show candidate therapeutic targets identified through multi‐omics studies.

**TABLE 3:** Potential targets identified through transcriptomic analysis and multi‐omics.

Method	Targets	Function	Supporting data	Model system
RNAseq	Midkine	Inhibits VIC calcification, reduces the expression of RUNX2 and ALP, involved in Notch signalling	[35]	Human aortic valves
	MAOA	Involved in apoptosis, associated with calcification	[45]	Human aortic valves
	CTHRC1	Involved in pro‐migratory pathways, inhibits collagen matrix synthesis, associated with calcification		
	Lumican	ECM protein involved in collagen fibril organisation, accelerates calcification	[46]	Human aortic valves
	ADIPOQ	Inhibits TNF‐α, decreasing inflammation	[84]	Human aortic valves
	TLR2	Accelerates inflammation	[85]	Human aortic valves
	CD86	Contributes to T‐cell activation and inflammation		
	TYROBP	Induces the production of pro‐inflammatory cytokines		
	THBS2, SPARC, COL1A2, COL1A1, SPP1, and CTGF	ECM regulators	[86]	Human aortic valves
	VCAM1, FHL2, RUNX1, TNFSF10, PLAU, SPOCK1, CD74, SIPA1L2, TRIB2, CXCL12	Involved in inflammation associated with AV stenosis	[87]	Human aortic valves
	VASP and ROCK1	Regulators of actin cytoskeleton	[3]	In vitro study using human valvular cells
	FAK, JAM2, claudin‐2, cadherins	Junctional proteins		
	MCU and MCUB	Regulation of mitochondrial calcium uptake	[88]	Human aortic valves
	SPP1	Involved in proliferation, apoptosis, and osteogenic differentiation	[49, 56]	Human aortic valves
	HMOX1	Osteogenic differentiation	[49]	Human aortic valves
	CD28	T cell activation		
	CircHIPK3	Protect against calcification by reducing calcium deposition and BMP‐2 expression	[55]	Human aortic valves
	miR‐182‐5p	Increase Wnt/β‐catenin activity		
	hsa‐piR‐25,624	Stabilises PCSK9, accelerating CAVD progression	[57]	High‐cholesterol diet–fed ApoE^−/−^ mice; human aortic valve samples
GWAS/TWAS	PALMD, TEX41, IL6, LPA, FADS	AVD risk loci	[59, 60, 61, 62, 64, 65]	Human samples
	PALMD	CAVD risk locus	[61]	Human samples
	PALMD, NAV1, PPRX1, TWIST1, MUC4, ATP13A3, ALDH1A2, LPL, LDLR, BCL10, RADA9, NRBP1, FES, AFAP1, LDLR, APOB and PCSK9	Aortic stenosis risk loci	[66, 89]	Human samples
Proteomics + RNAseq	ANXA1	Contributes to calcification	[77]	Human aortic valves
	ApoC‐III	Promotes calcification through mitochondrial dysfunction	[79]	Human aortic valves
	FNDC1	Involved in osteoblast activity	[82]	Human aortic valves

*Note:* Potential therapeutic targets related to AV disease through transcriptomic and multi‐omics approaches. For each method listed, relevant genes and their functions are described, emphasising their roles in processes such as associated with AV pathology. Additionally, the genetic factors associated with AV disease risk are documented.

## 4 Conclusions and Perspectives

Aortic valve disease remains a global health challenge with no approved pharmacological treatments, the only current solution being valve replacement through surgery. This underscores the urgent need to better understand the complex pathophysiology of the valve to develop targeted, non‐invasive therapies. Advances in high‐throughput technologies, including microarrays, bulk RNA and single‐cell RNA sequencing, have significantly expanded our understanding of aortic valve physiology and pathology. These methods have uncovered the remarkable cellular heterogeneity of the valve, revealed the plasticity of valvular cells, and elucidated key mechanisms of cellular crosstalk that maintain valvular homeostasis. Disruption of these mechanisms contributes to disease progression.

While scRNAseq has provided unprecedented insights into the cellular complexity of heart valves, it also presents technical limitations, including dissociation bias that can skew cell‐type representation, potentially underrepresenting fragile or difficult‐to‐dissociate cell populations and ‘dropout events’ that may obscure low‐expressed transcripts. Integrating scRNAseq with complementary techniques, such as spatial transcriptomics and proteomics, can mitigate these limitations and offer a more comprehensive picture of valve biology and disease mechanisms.

Further research should focus on the maintenance and repair mechanisms of the healthy adult valve and their response to injury. In particular, the role of long underappreciated VECs require further exploration. Specifically, it is important to understand VECs transcriptional profiles associated with early CAVD. Transcriptomic studies continue to uncover important VECs contribution to CAVD progression and further omics‐based investigations are needed to further advance the understanding of their contribution.

Defining the cellular heterogeneity of the human AV and the specific molecular signatures associated with CAVD progression is essential for developing novel cellular‐specific and mechanism‐based therapies. The role of tissue‐resident macrophages in valve regeneration and repair and their involvement in disease remain to be elucidated.

Diabetes is an established risk factor for accelerated AVD, yet the molecular mechanisms driving this relationship are poorly understood. Our recent studies demonstrate that nanocarrier‐based therapies may offer a promising approach to block CAVD progression in diabetic models [90, 91, 92].

Emerging omics approaches offer powerful tools to dissect the biological complexity of AVD disease. They provide insights into cellular diversity, cellular interactions, molecular signatures and regulatory mechanisms and help identify potential therapeutic targets. Several candidates have been proposed in the regulation of aortic valve pathology, highlighting key processes such as calcification, inflammation and ECM remodelling. Potential targets include midkine (protective, inhibiting VIC calcification), Lumican and ApoC‐III (pro‐calcific drivers), ADIPOQ, TLR2, CD86, TYROBP (inflammatory modulators), THBS2, SPARC and SPP1 (ECM regulators), VASP and ROCK1 (cytoskeletal regulators). However, translating these findings into therapies requires further functional validation, especially in physiologically relevant in vitro and animal models, and ultimately in large‐scale clinical trials.

Functional validation of GWAS‐identified genetic loci (e.g., PALMD, TEX41, IL6, LPA and FADS) and epigenetic modifications is ongoing. Integration of multi‐omics data remains complex and can hinder timely identification of actionable targets.

Access to non‐diseased valve tissue samples remains a limiting factor for comparative analyses between healthy and diseased tissues, limiting the ability to identify specific pathophysiological changes associated with AVD. Increasing collaborations with transplant centres and biobanks could help expand available resources and enhance the accuracy of disease‐specific insights.

Despite current challenges, omics approaches are poised to transform our understanding of CAVD. Continued exploration of these technologies will improve disease modelling, diagnosis, and lead to the development of personalised, mechanism‐based interventions that improve outcomes for patients with CAVD.

## Author Contributions

**Monica Madalina Tucureanu:** conceptualization (lead), writing – original draft (lead). **Ileana Manduteanu:** writing – review and editing (lead).

## Conflicts of Interest

The authors declare no conflicts of interest.

## Data Availability

The authors have nothing to report.
